# The relative age effect among Malaysian university athletes: a cross-sectional survivorship analysis with exploratory AI-based movement screening

**DOI:** 10.3389/fspor.2026.1884958

**Published:** 2026-07-22

**Authors:** Haashwein Moganan, Mohansundar Sankaravel, Gunathevan Elumalai, Jin Seng Thung, Jianhong Gao

**Affiliations:** 1 Department of Coaching Science, Faculty of Sport Science and Coaching, Sultan Idris Education University, Tanjong Malim, Perak Darul Ridzuan, Malaysia; 2 Department of Health Science, Faculty of Sport Science and Coaching, Sultan Idris Education University, Tanjong Malim, Perak Darul Ridzuan, Malaysia; 3 Research and Innovation Division, National Sports Institute of Malaysia, Kuala Lumpur, Malaysia; 4 Faculty of Sports and Exercise Science, University of Malaya, Kuala Lumpur, Malaysia

**Keywords:** athlete development, biomechanical screening, birth quartile, markerless motion capture, talent identification

## Abstract

The relative age effect (RAE) is defined as the overrepresentation of athletes born early in the selection year and is well documented in talent identification. However, whether the selection pressures that produce the RAE leave a detectable biomechanical signature in the athletes who reach elite level remains unresolved. This cross-sectional study examined the relative age effect among national-level Malaysian university athletes and, as a secondary exploratory question, whether any biomechanical loading signature accompanied it. Specifically, it tested whether (i) selection asymmetry consistent with the RAE was present, (ii) the RAE differed by gender, and (iii) AI-derived loading-risk indices differed across birth quartiles. A total of 170 athletes (116 males, 54 females; *M* = 18.6, *SD* = 0.7 years) were assessed using the Holomotion markerless motion capture system, generating four pre-specified outcomes: a composite total movement score, an exercise risk score, a joint pain risk index, and a ligament strain risk index (the latter two reflecting modelled tissue-level loading rather than clinical injury events). The birth quartiles were derived from national identification numbers using a 1st January cut-off. A significant RAE was observed overall [*χ*^2^(3) = 11.74, *p* = .008, w = 0.26] and in males [*χ*^2^(3) = 8.55, *p* = .036, w = 0.27; Q1:Q4 = 2.06], but not in females [*χ*^2^(3) = 3.78, *p* = .286]. No loading-risk index differed across quartiles, either as continuous scores (Kruskal–Wallis, all *p* ≥ .720; *η*^2^ ≈ 0) or as binary high-risk classifications (chi-square, all *p* ≥ .660; Cramér's V ≤ 0.10). The selection-related birthdate asymmetry was therefore not accompanied by detectable differences in AI-derived biomechanical loading estimates, a pattern consistent with a survivorship interpretation.

## 1 Introduction

Musculoskeletal injury is a primary determinant of athlete availability, performance development, and long-term participation in collegiate and emerging-adult sport (1). Adolescent and emerging-adult cohorts in particular show high injury burden during competitive seasons, with overuse and lower-extremity injuries dominating epidemiological surveys (2, 3). Annual age-grouping with a fixed cut-off date is the structural backbone of nearly every sport pathway worldwide, and this practice produces the relative age effect (RAE): the systematic overrepresentation of athletes born early in the selection year relative to their later-born peers (4, 5). Whether selection-related birthdate asymmetry intersects with objectively assessed musculoskeletal risk in the athletes who survive into pathway-level competition is a question of direct relevance to both talent-identification practice and athlete welfare. Despite four decades of RAE research and a parallel surge in AI-augmented biomechanical screening, these two lines of evidence have developed independently and have rarely been brought into direct empirical dialogue (6, 7).

The body of knowledge in relative age effects is now firmly established. Recent analyses spanning four decades of Ballon d'Or nominees, multi-sport Youth Olympic Games competitors, and Olympic karate athletes have consistently shown overrepresentation of relatively older athletes at every selection level (5, 8, 9). Female effects are typically smaller in magnitude but directionally similar to those reported in males (10, 11). The injury consequences of this asymmetry, however, remain contested. A cohort of 1,997 paediatric athletes reported significantly more injuries among relatively older children, a pattern attributed to early-maturing players carrying larger training loads (12). In direct tension with this, a growing body of work supports an underdog or survivorship hypothesis as late-born athletes who reach elite levels represent a self-selected sub-population whose technical, tactical, and perceptual-cognitive attributes have compensated for an early maturity disadvantage (13, 14). Recent large-scale longitudinal evidence continues to support this survivorship reading: a 44-year analysis of 10,485 NHL-drafted players found that late-born athletes who overcame draft under-representation demonstrated significantly faster league entry and higher career permanence than their relatively older counterparts, consistent with compensation operating through non-physical selection pathways (15). Qualitative systematic-review evidence further indicates that long-term individual performance is in fact more often associated with reversed RAE patterns than with the early-selection bias itself, although world-class track and field data show that the 1st quartile birth advantage can persist into adulthood in some events (16, 17). A recent study among Malaysian collegiate track and field athletes likewise found that RAE and injury prevalence co-occur in event-specific rather than uniformly graded ways (18). However, whether such residual asymmetry is detectable in objective biomechanical indices rather than self-reported injury counts has not been examined.

The theoretical mechanism by which RAE could translate into a detectable biomechanical signature operates through two well-established pathways: biological maturation and cumulative training exposure. A recent scoping review of elite youth pathway athletes established that maturity status is associated with increased injury incidence and burden, and that more rapid somatic growth links directly to greater injury susceptibility, particularly for lower-limb tissues (19). Training load itself is the proximal determinant of tissue-level exposure and injury occurrence (20, 21). RAE selection could plausibly leave one of two competing biomechanical imprints on surviving athletes, since chronological age partially indexes biological maturity and training exposure accumulates differentially for early- and late-born athletes across the selection pathway. Under a cumulative-load account, late-born athletes at elite level would carry residual biomechanical risk from the prolonged compensatory training required to remain in the pathway. Under a survivorship account, late-born athletes reach elite level primarily through technical, tactical, and psychological compensation, and no biomechanical difference would be expected between quartiles. Empirical evidence favours the survivorship account: a 10-year retrospective analysis of an elite Scottish soccer academy found that anthropometric and physical performance variables did not reliably predict successful contract award despite a strong 1st quartile bias at recruitment, and academy-cohort work has directly linked late-maturity status to superior self-regulation skills consistent with non-physical compensation (22, 23). The two accounts therefore make directionally opposing, empirically testable predictions in surviving cohorts.

Objective movement screening now offers a technological pathway to test these competing predictions directly, moving beyond self-reported or clinically recorded injury counts to standardised biomechanical estimates. Markerless motion capture (MMC) reliability studies report good-to-excellent intraclass correlations for lower-limb and trunk kinematics during functional tasks, with comparable repeatability for algorithm-derived motion-health screening scores generated by camera-based MMC systems (24–26). Multi-sport reviews indicate that AI-augmented MMC platforms are sufficiently mature for scalable field-based screening across heterogeneous athletic populations (6, 7). From a theoretical standpoint, contemporary developmental-systems models conceptualise the RAE as the emergent product of interacting individual, task, and environmental constraints rather than a single unidirectional bias, implying that biomechanical consequences if present would themselves be context-dependent rather than uniformly graded (27). The specific gap addressed here is therefore three-fold. First, the RAE evidence in Southeast and East Asian collegiate sport remains relatively limited (18, 28). Furthermore, the existing RAE–injury literature relies almost exclusively on self-reported or clinically recorded injury counts, which conflate biomechanical predisposition with exposure-driven incidence (12). Consequently, AI-derived biomechanical screening has not been deployed as a measurement bridge between RAE selection and downstream movement quality previously.

Therefore, the aim of the present study was to examine, in a cohort of national-level Malaysian university athletes, (i) whether selection asymmetry consistent with the RAE was present, and (ii) whether the RAE differed by gender, given consistent evidence that RAE magnitude is typically stronger in male than female athletic populations. As a secondary exploratory aim, the study additionally examined (iii) whether pre-specified AI-derived loading-risk indices differed across birth quartiles, with the exploratory framing reflecting the absence of independent criterion validation for the system. Drawing on the theoretical framework outlined above, and consistent with the increasing empirical support for the survivorship account, it was hypothesised that a RAE would be detectable as overrepresentation of 1st quartile-born athletes but that biomechanical loading indices would not differ across birth quartiles, since athletes who reach this competitive level are predicted to have done so primarily through non-physical compensation rather than through a biomechanically distinctive selection pathway (13, 14).

## 2 Materials and methods

### 2.1 Study design

A cross-sectional observational study was conducted from 9 to 15 September 2025 at the Faculty of Sport Science and Coaching, Sultan Idris Education University, Malaysia. Reporting follows the Strengthening the Reporting of Observational Studies in Epidemiology (STROBE) statement (29). The protocol was approved by the Sultan Idris Education University Research Ethics Committee (Reference Number: 25-0816-01) and conducted in accordance with the Declaration of Helsinki. Written informed consent was obtained from all participants prior to data collection.

### 2.2 Participants

Participants were recruited from the Malaysia Juara Programme, a national initiative hosted at the university for student-athletes competing at state, national, and international levels. Inclusion required current enrolment in the study programme, active competition at minimum state level, and absence of acute musculoskeletal injury. Exclusion criteria were post-surgical conditions, acute illness, or any medical contraindication to physical assessment. An initial pool of (*N* = 180) screening records was retrieved from the institutional dataset. Records were excluded if data on the national identification number, sport modality, or gender were incomplete or missing and could not be reconciled against institutional records. Ten records (5.6%) met one or more of these criteria and were excluded, yielding a final analysed cohort of (*N* = 170) athletes (*n* = 116 male; *n* = 54 female), aged (*M* = 18.6, *SD* = 0.7 years). Athletes in this cohort meet the criteria for Tier 3 (Highly Trained/National Level) under the participant classification framework now widely adopted in sports science research (30), competing at recognised state and national fixtures with structured multi-session weekly training. Sampling was by convenience within the host institution.

### 2.3 Sample size

This analysis used a convenience sample drawn retrospectively from an existing institutional movement-screening database, and *a priori* sample size calculation was therefore not feasible. A *post-hoc* sensitivity analysis was conducted using G*Power 3.1 (31). For the chi-square goodness-of-fit test (df = 3, *α* = .05, 1−*β* = .80, *N* = 170), the minimum detectable effect size was Cohen's w = 0.234, indicating that the present sample provides 80% power to detect small-to-medium birth-quartile asymmetries. For Kruskal–Wallis comparisons of four groups (k = 4, df = 3, *α* = .05, 1−*β* = .80), the minimum detectable effect was *η*^2^ = 0.058 (medium effect by Cohen's conventions). Findings should be interpreted within these power constraints.

### 2.4 Birth quartile determination

Each participant's date of birth was extracted from their Malaysian national identification card, the YYMMDD-encoded national registration number issued under the Malaysian National Registration Act 1959. Birth quartiles were assigned using a 1 January cut-off, consistent with prior RAE work in Malaysian collegiate athletes and with the methodological conventions adopted by the broader RAE literature where Q1 included athletes born in January–March, Q2 in April–June, Q3 in July–September, and Q4 in October–December (5, 8, 18).

### 2.5 AI-based movement screening protocol

Movement quality and injury risk were assessed using the Holomotion markerless motion capture system (Beijing Xinqing Taike Sports Technology, Beijing, China). The system uses a depth-sensing camera array with proprietary computer-vision and machine-learning algorithms to reconstruct three-dimensional joint kinematics during functional movement tasks. For each movement the system extracts joint range-of-motion, starting value, and maximum deviation parameters; aggregates these into regional sub-scores (neck, shoulder and upper limb, torso, pelvis, lower limb) across range-of-motion, stability, and symmetry domains; and derives composite scores together with condition-specific risk indices reported on a banded scale (low = 0–15, medium = 16–55, high = 56–100). The eleven movements, displayed sequentially, were neck forward flexion, neck extension, left neck lateral flexion, right neck lateral flexion, wall angel, upright rotation–left, upright rotation–right, single leg raises, overhead squat, side lunges, and lunge squats. The 11 movements were pre-set in the system and were not selected by the research team. These movements overlap substantially with two established functional-screening frameworks. Cervical flexion, extension, rotation, overhead squat, single-leg stance, and trunk rotation correspond to Top Tier Movements of the Selective Functional Movement Assessment (SFMA); the overhead squat, in-line lunge, shoulder mobility, and active straight leg raise mirror items in the Functional Movement Screen (FMS) (32, 33). Both frameworks have prospective injury-risk evidence; for example, pain during the SFMA overhead squat and single-leg stance movements increased the odds of subsequent lower-extremity time-loss injury 1.76-fold in a prospective military cohort (*n* = 480) (34). Each of the eleven movements was performed three times; for each movement the system retained the single best-performing repetition according to its internal scoring algorithm, and this value was used for analysis.

The test–retest reliability of the Holomotion system has been reported among healthy participants (24). Within-day reliability was excellent for mobility and pelvis indices (ICC > 0.90) and good for most remaining domain variables (ICC = 0.83–0.89), with moderate reliability for symmetry, neck pain, and shoulder pain; between-day reliability was moderate for scoliosis and symmetry and poor for shoulder pain (ICC = 0.304). The study reported reliability only, not concurrent validity against a criterion measure. However, to date no independent criterion-validity study comparing the Holomotion system against gold-standard marker-based three-dimensional motion capture has been published. Analogous evidence from other markerless motion capture platforms provides contextual but not direct support for the present measurement framework. A deep-learning markerless system evaluated against marker-based motion capture during the overhead squat reported acceptable concurrent validity for lower-limb kinematics (25), and a three-dimensional markerless system used for motion-health screening scores demonstrated good session-to-session repeatability across functional movements (26). Therefore, the AI-derived output indices should be interpreted as exploratory rather than confirmatory measures as the concurrent validity remains empirically unestablished at the time of writing.

Each participant first completed anthropometric measurements: standing height to the nearest 0.1 cm using a calibrated portable stadiometer (Model 213, SECA GmbH & Co. KG, Hamburg, Germany), and body mass to the nearest 0.1 kg using a multi-frequency body composition analyser (Model BF-679W, TANITA Corporation, Tokyo, Japan). The anthropometric values, age, and gender were entered into the Holomotion application interface directly as required calibration inputs. The Holomotion depth-sensing camera unit was mounted 1 m above ground level facing the participant, with an audio speaker positioned to one side to deliver the system's movement-cue prompts. Each participant was then positioned at a standardised distance of approximately 2 m facing the depth-sensing camera unit. Once skeletal calibration was confirmed by the system, the assessor initiated the protocol.

### 2.6 Outcome variables and terminological note

Based on the nine indices generated by the system, four were pre-specified as relevant to the RAE-injury hypothesis. The Total Movement Score (0–100, higher = better movement quality) and the Exercise Risk Score were retained as composite indices of overall movement quality and global injury vulnerability respectively. The Joint Pain risk index and the Ligament Strain risk index were retained as secondary outcomes given their direct relevance to the cumulative-load mechanism by which RAE-related selection bias could plausibly leave a residual biomechanical signature in surviving athletes. Furthermore, the joint loading patterns reflect chronic training-exposure history, while ligamentous integrity is sensitive to repeated submaximal stress accumulated across years of competitive participation (20). Selection of these specific outcomes is also consistent with prospective biomechanical evidence linking knee-joint loading and ligamentous-sprain proxies to subsequent lower-limb injury in athletic populations (35, 36). The remaining five region-specific scores (neck pain, shoulder pain, scoliosis, lumbar pain, ankle sprain) were not pre-specified as central to the RAE-injury hypothesis and were excluded from confirmatory testing to limit multiple comparisons.

A terminological clarification is warranted at the outset. Within the Holomotion framework, the “Ligament Strain risk index” denotes a biomechanical estimate of ligamentous tissue deformation under modelled joint loading. The term “strain” is used here in its engineering sense as a measure of relative tissue elongation (*ε* = *Δ*L/L_0_) and is not a clinical prediction of ligament sprain incidence (37). Likewise, the “Joint Pain risk index” is an algorithmically derived estimate of predisposition to develop joint pain under continued loading, rather than a measurement of currently reported arthralgia. These distinctions matter because clinical sport medicine reserves “sprain” for ligamentous injury events and “strain” for muscular/tendinous events; the Holomotion outputs sit within the biomechanical-modelling vocabulary rather than the clinical-injury vocabulary.

### 2.7 Statistical analysis

Analyses were performed in IBM SPSS Statistics version 26.0 (IBM Corporation, Armonk, NY, USA). Descriptive statistics (mean, standard deviation, median, 25th and 75th percentiles, minimum, maximum) were computed for the four AI-derived outcomes. Normality was assessed using the Shapiro–Wilk test; non-parametric methods were used for all inferential comparisons given non-normal distributions across all four outcomes (all *p* < .001).

Three pre-specified analytic steps tested the study hypotheses. First, a chi-square goodness-of-fit test compared the observed birth-quartile distribution against a uniform expected distribution (25% per quartile); robustness was checked by repeating the test using a days-per-quarter weighted distribution (Q1 = 24.66%, Q2 = 24.93%, Q3 = 25.21%, Q4 = 25.21%) (4). The same goodness-of-fit test was repeated separately for males and females. Effect sizes were quantified using Cohen's w (small = 0.10, medium = 0.30, large = 0.50) and the Q1 to Q4 ratio (38). Second, Kruskal–Wallis tests compared each of the four AI-derived outcomes across birth quartiles, with *η*^2^ as the effect size estimate. Third, athletes were classified as high-risk on each outcome based on within-sample 75th percentile cut-off. The chi-square tests of independence were then employed to examine whether birth-quartile distribution differed by high-risk status. The Cramér's V was used to estimate the effect size. Statistical significance was set at *α* = .05 (two-tailed) for all tests.

## 3 Results

### 3.1 Participant characteristics

The analysed cohort comprised 170 athletes (116 males, 54 females; *M* = 18.6, *SD* = 0.7 years). The birth-quartile distribution was Q1 = 51 (30.0%), Q2 = 37 (21.8%), Q3 = 55 (32.4%), Q4 = 27 (15.9%). The cohort spanned 27 sport modalities across six broad categories, with team sports comprising the largest sub-cohort (*n* = 103; 60.6%) and individual sports the second largest (*n* = 35; 20.6%); the full sport distribution is presented in Table 1. The descriptive statistics for the four AI-derived outcomes are presented in Table 2 below.

**Table 1:** Sport distribution of the analysed cohort (*N* = 170).

Sport category	Sport	*n*	Total *n*	%
Team sports	Hockey	22	103	60.6
	Rugby	20		
	Softball	11		
	Football	10		
	Handball	9		
	Sepak Takraw	9		
	Volleyball	7		
	Cricket	5		
	Netball	4		
	Futsal	3		
	Basketball	1		
	Lawn Bowls	1		
	Dodgeball	1		
Individual sports	Athletics	28	35	20.6
	Cycling	5		
	Swimming	1		
	Kayaking	1		
Combat sports	Silat	8	11	6.5
	Taekwondo	3		
Precision sports	Archery	5	11	6.5
	Bowling	4		
	Golf	1		
	Pétanque	1		
Racquet sports	Badminton	3	7	4.1
	Squash	3		
	Tennis	1		
Aesthetic sports	Gymnastics	3	3	1.8
Total		**170**	**170**	**100**.**0**

**Table 2:** Descriptive statistics for the four AI-derived outcomes (*N* = 170).

Outcome	Mean (SD)	Median (P25–P75)	Min–Max	P75 cut-off
Total Movement Score	76.28 (4.02)	76.45 (74.17–78.80)	59.50–85.02	78.80
Exercise Risk Score	15.87 (2.91)	15.71 (14.04–17.36)	9.74–28.58	17.36
Joint Pain risk index (%)	19.75 (6.43)	18.79 (15.62–23.64)	7.00–42.54	23.64
Ligament Strain risk index (%)	24.17 (4.42)	23.97 (21.26–26.57)	14.83–42.35	26.57

Higher Total Movement Scores indicate better movement quality; higher percentages on the remaining outcomes indicate greater estimated loading risk. Joint Pain and Ligament Strain risk indices reflect modelled tissue-level loading rather than clinically observed injury events.

SD, standard deviation; P25, 25th percentile; P75, 75th percentile.

### 3.2 Relative age effect

The chi-square goodness-of-fit test indicated a significant deviation from a uniform birth-quartile distribution at the cohort level [*χ*^2^(3) = 11.74, *p* = .008, Cohen's w = 0.26, Q1:Q4 ratio = 1.89]. The same test using a days-per-quarter weighted expected distribution yielded a near-identical result [*χ*^2^(3) = 11.96, *p* = .008], confirming the result was robust to baseline choice. Gender-stratified analyses showed a significant RAE in males [*n* = 116; *χ*^2^(3) = 8.55, *p* = .036, w = 0.27, Q1:Q4 = 2.06], but not in females [*n* = 54; *χ*^2^(3) = 3.78, *p* = .286, w = 0.27, Q1:Q4 = 1.60]. Birth-quartile observed and expected counts are presented in Table 3.

**Table 3:** Birth-quartile distribution and chi-square goodness-of-fit results.

Group	*N*	Q1, *n* (%)	Q2, *n* (%)	Q3, *n* (%)	Q4, *n* (%)	*χ*^2^ (3)	*p*	*w*
Overall	170	51 (30.0)	37 (21.8)	55 (32.4)	27 (15.9)	11.74	.008	0.26
Males	116	35 (30.2)	27 (23.3)	37 (31.9)	17 (14.7)	8.55	.036	0.27
Females	54	16 (29.6)	10 (18.5)	18 (33.3)	10 (18.5)	3.78	.286	0.27

χ^2^, chi-square goodness-of-fit statistic against uniform expected distribution; w, Cohen's w (0.10 = small, 0.30 = medium, 0.50 = large).

### 3.3 Birth quartile and AI-derived loading-risk indices

**Figure 1:**
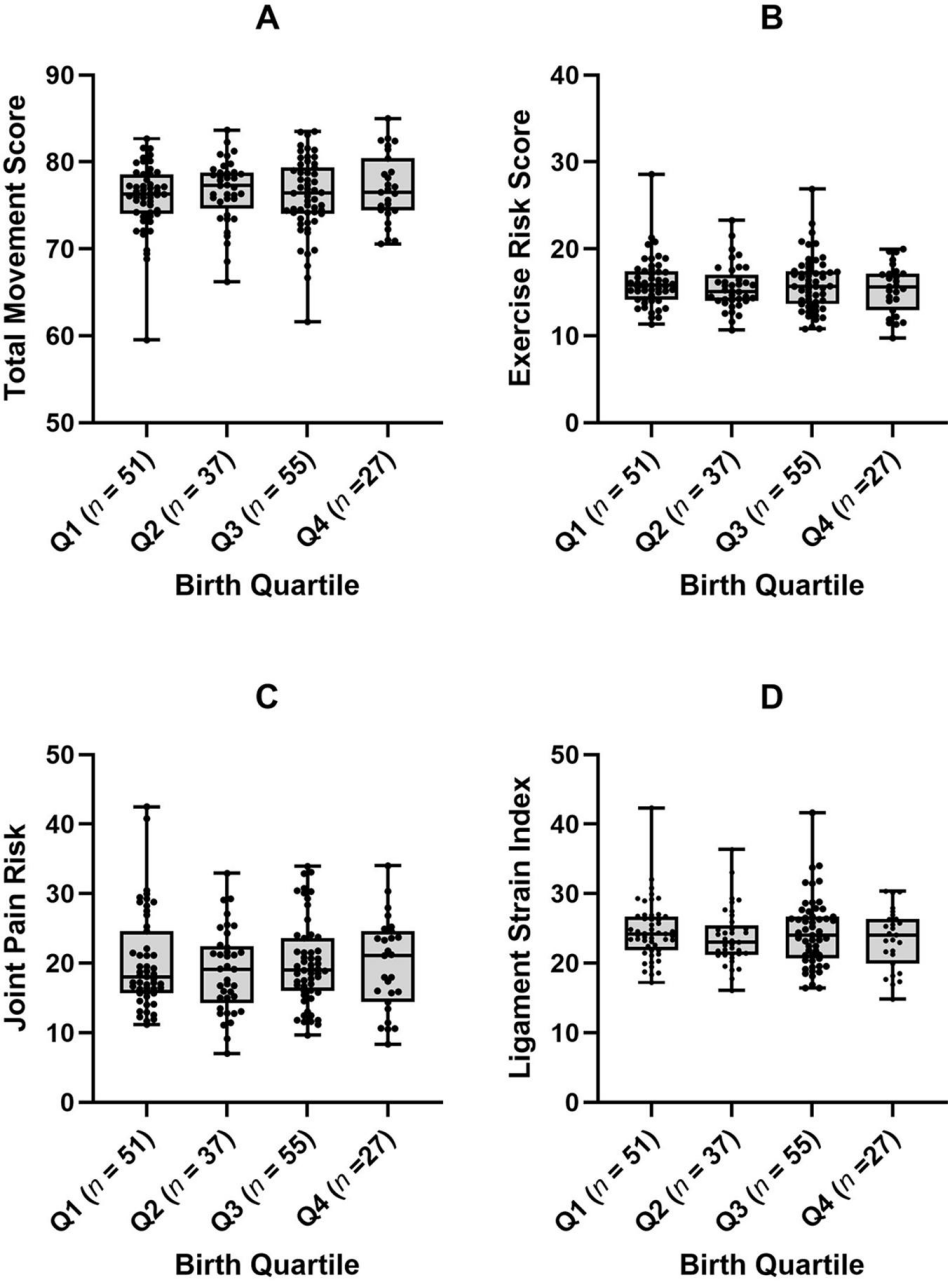
Distribution of the four pre-specified holomotion-derived indices across birth quartiles (*N* = 170). **(A)** Total Movement Score; **(B)** Exercise Risk Score; **(C)** Joint Pain risk index; **(D)** Ligament Strain risk index. Boxes show the median and interquartile range; whiskers span the full range of observed values; each point represents one individual athlete. Q1 = January–March, Q2 = April–June, Q3 = July–September, Q4 = October–December. The substantial overlap of distributions across quartiles in all four panels corresponds to the non-significant Kruskal–Wallis results reported in Table 4 (all *p* ≥ .720).

The Kruskal–Wallis tests revealed no differences in any of the four AI-derived outcomes across birth quartiles: Total Movement Score [H(3) = 1.19, *p* = .755, *η*^2^ ≈ 0], Exercise Risk Score [H(3) = 1.18, *p* = .757, *η*^2^ ≈ 0], Joint Pain risk index [H(3) = 0.50, *p* = .918, *η*^2^ ≈ 0], and Ligament Strain risk index [H(3) = 1.36, *p* = .715, *η*^2^ ≈ 0]. Mean ranks across the four quartiles spanned no more than 11 ranks (out of 170 possible) on any outcome, indicating substantive equivalence rather than borderline non-significance. Median values and inferential statistics are presented in Table 4, below. The absence of between-quartile separation across all four indices is shown in Figure 1.

**Table 4:** AI-derived outcomes by birth quartile (Kruskal–Wallis).

Outcome	Q1 (*n* = 51) Mdn	Q2 (*n* = 37) Mdn	Q3 (*n* = 55) Mdn	Q4 (*n* = 27) Mdn	H(3)	*p*	*η* ^2^
Total Movement Score	76.28	76.83	76.34	76.95	1.19	.755	≈0
Exercise Risk Score	16.04	15.34	15.85	15.34	1.18	.757	≈0
Joint Pain risk index	18.81	18.34	19.27	18.79	0.50	.918	≈0
Ligament Strain risk index	24.40	23.50	23.97	23.50	1.36	.715	≈0

Median values are illustrative; full distributional comparison is reflected in the Kruskal–Wallis H statistic.

Mdn, median; η^2^, eta-squared effect size.

### 3.4 Binary high-risk classification

To complement the distribution-level analyses, athletes were classified as high-risk on each outcome using the within-sample 75th percentile cut-off (Total Movement Score ≥ 78.80, Exercise Risk Score ≥ 17.36, Joint Pain risk index ≥ 23.64, Ligament Strain risk index ≥ 26.57). Chi-square tests of independence revealed no associations between birth quartile and high-risk classification on any outcome: HR-Total [*χ*^2^(3) = 1.58, *p* = .664, V = 0.10]; HR-Exercise Risk [*χ*^2^(3) = 0.13, *p* = .989, V = 0.03]; HR-Joint Pain [*χ*^2^(3) = 0.59, *p* = .898, V = 0.06]; HR-Ligament Strain [*χ*^2^(3) = 0.49, *p* = .921, V = 0.05]. No expected cell count fell below 5 in any of the four contingency tables, confirming the validity of the chi-square approximation. The counts are presented in Table 5 below.

**Table 5:** High-risk classification by birth quartile.

Outcome	Q1 (*n* = 51)	Q2 (*n* = 37)	Q3 (*n* = 55)	Q4 (*n* = 27)	χ^2^ (3)	*P*	*V*
HR Total Movement	10 (19.6%)	9 (24.3%)	16 (29.1%)	8 (29.6%)	1.58	.664	0.10
HR Exercise Risk	13 (25.5%)	9 (24.3%)	14 (25.5%)	6 (22.2%)	0.13	.989	0.03
HR Joint Pain	13 (25.5%)	8 (21.6%)	13 (23.6%)	8 (29.6%)	0.59	.898	0.06
HR Ligament Strain	13 (25.5%)	8 (21.6%)	15 (27.3%)	6 (22.2%)	0.49	.921	0.05

HR, high-risk classification (≥within-sample 75th percentile on the relevant outcome); V, Cramér's V (0.10 = small, 0.30 = medium, 0.50 = large).

## 4 Discussion

This study provides the first examination, in a Southeast Asian university cohort, of whether the relative age effect, a robust selection phenomenon, is accompanied by a detectable biomechanical signature in the surviving athletes. A relative age effect was clearly evident at the cohort level and among male athletes, consistent with international literature (5, 9). However, despite this demonstrable selection asymmetry, none of the four pre-specified AI-derived loading-risk indices differed across birth quartiles, in either continuous or categorical analyses. The selection bias was real; however, the biomechanical trace, as captured by modelled tissue-level loading estimates, was not detectable. The cohort-level Q1:Q4 ratio of 1.89, and the male-specific ratio of 2.06, sit comfortably within the range reported for collegiate and pre-elite male cohorts internationally (5, 9).

The male–female asymmetry — significant in males but non-significant in females despite an identical effect size (w = 0.27 in both groups) — is also consistent with international evidence of weaker female RAEs that more often reflect statistical power than biological absence. A meta-analytic pooling across 308 female samples spanning from 1984 to 2016 confirmed a similar small-to-moderate pooled effect, with explicit emphasis on power-related null findings in smaller cohorts (11). Small female RAEs are frequently reported yet often fail to reach significance in modestly sized samples, a pattern consistent with the non-significant female result observed here despite an effect size equivalent to that in males (w = 0.27) (10). A second feature of the present distribution warrants explicit attention. Q3 over-representation (32.4%) exceeded that of Q1 (30.0%), with Q2 (21.8%) sitting unexpectedly below Q3, departing from the monotonic Q1 > Q2 > Q3 > Q4 gradient that characterises classical maturation-driven RAE (4). The significant goodness-of-fit result therefore reflects birth-quartile non-uniformity that is only partially consistent with a standard RAE pattern: the Q1 over-representation and Q4 deficit (15.9%) align with selection asymmetry, but the Q3 inflation does not, and may reflect cohort-specific recruitment cycles within the Malaysia Juara Programme, school-year enrolment patterns under the Malaysian academic calendar, or institutional intake patterns at the host university. A comparable Q3 over-representation has been documented in a prior Malaysian collegiate track and field cohort, suggesting a recurring rather than cohort-specific pattern (18). Although Malaysian schooling and MSSM selection share a 1st January cut-off, tertiary institution intake operates across multiple windows (March and September), which may introduce overlapping selection pathways that depart from the classical monotonic Q1 > Q2 > Q3 > Q4 gradient. The Q1:Q4 ratio of 1.89 remains within the range reported for collegiate male cohorts internationally, but the non-monotonic distribution should be replicated in independent Malaysian collegiate samples before the present pattern is treated as a stable feature of the population.

The key inferential value of this design lies not in whether asymmetry is present in an injured sample, but in whether selection-related birthdate asymmetry leaves a detectable biomechanical signature among the survivors who reach the sampled competitive level (4, 27). As outlined in the theoretical framework, survivorship and cumulative-load accounts make directionally opposing predictions in this population: the former predicts biomechanical equivalence across birth quartiles, the latter predicts elevated loading estimates in later-born athletes. A null result in a healthy survivor cohort is therefore not a foregone conclusion but the specific outcome predicted by one account and contradicted by the other. The present findings align with the survivorship prediction: none of the four AI-derived loading indices showed a birth-quartile signal, with mean rank differences spanning no more than 11 ranks out of 170 possible on any outcome, *η*^2^ values rounding to zero, and Cramér's V values falling uniformly below the small-effect threshold. Q4 athletes neither showed elevated estimated loading risk nor reduced loading risk relative to their earlier-born peers.

This pattern is most parsimoniously interpreted as biomechanical-loading equivalence across birth quartiles, consistent with late-born athletes reaching elite level by compensating for any early maturity disadvantage through technical, tactical, perceptual, or psychological development, rather than by being filtered through a biomechanically distinctive selection process (13, 39, 40). Direct empirical support for this developmental pathway has been reported in academy soccer cohorts, where later-maturing players showed superior self-regulation skills consistent with the underdog hypothesis (23), and in pathway cohorts where relatively younger players, despite early physical disadvantage, more often reached the elite level (41). A complementary explanation is that biological maturity, rather than chronological birth date, may be the more proximal driver of physical-performance differences in pathway athletes; in a sample of *N* = 199 male academy players aged 8.1–18.9 years, maturity status had a substantially greater association with sprint and jump performance than relative age (42). Consequently, by the time athletes reach a Tier 3 cohort, the physical-maturation differences that separate Q1 from Q4 in early adolescence have largely closed. Importantly, the cumulative-load mechanism that would predict differential joint and ligamentous loading by relative age operates through training exposure (20, 21), and cross-sectional snapshots of survivors capture neither the longitudinal exposure history nor the clinical injury events that the modelled risk indices are intended to anticipate. The present null finding is therefore a statement about what cross-sectional AI-derived loading estimation can detect in a survivor cohort, not a refutation of the underlying training-load mechanism — a constraint consistent with broader appraisals of four decades of relative age research (43).

This study has several methodological strengths. It draws on a moderately large cohort, employs an AI-derived measurement framework that is independent of self-report, and follows a pre-specified analytical pipeline in which the central hypothesis is tested through both continuous and binary approaches. However, some limitations remain. The cross-sectional design precludes causal inference about whether RAE-related selection bias affects subsequent injury occurrence; only prospective injury surveillance can address that question, and the present null result should be interpreted as evidence about cross-sectional biomechanical loading estimation rather than about longitudinal injury risk. Furthermore, the female subsample (*n* = 54) was underpowered to detect the effect that reached significance in males, despite yielding an identical effect-size estimate (w = 0.27). The non-significant female result is therefore most plausibly attributable to a Type II error rather than a genuine absence of effect, with the equivalent point estimate indicating that the effect was present but the subsample lacked sufficient power to detect it.

Additionally, the most important caveat for the interpretation of the null findings is that the Holomotion system's outputs are biomechanical loading estimates, not validated clinical injury predictions. A related and equally consequential limitation concerns the measurement instrument itself. While the Holomotion system has published test–retest reliability evidence (24), no independent study has established its concurrent validity against gold-standard marker-based motion capture. The reliability and validity evidence from other markerless platforms (25, 26) provides only contextual support because such findings do not transfer automatically across systems with different hardware, algorithms, and derived-metric pipelines. The “ligament strain risk index” models tissue-level deformation under predicted load and is not a prospectively validated predictor of clinical sprain incidence. The “joint pain risk index” similarly models loading-related predisposition rather than reported arthralgia.

Furthermore, screening tests tend to perform poorly when injury risk is multifactorial and emerges from non-linear interactions between athlete, training load, and contextual factors (44, 45). The predictive utility of even the most widely used field tools remains contested and machine-learning approaches to injury prediction have been similarly limited by methodological heterogeneity and small sample sizes in published validation studies (32, 33). Additionally, biopsychosocial frameworks indicate that mental, social, and load-related variables jointly explain injury risk in adolescent elite athletes more than biomechanical variables alone (46). Independent prospective validation of the specific Holomotion risk indices against actual injury incidence has yet to be published. Additionally, the 27-sport composition precluded sport-stratified RAE testing; the observed male effect may be driven disproportionately by team-sport sub-cohorts, and sport-specific confirmation is warranted (16). Furthermore, the sampling was by convenience within a single Malaysian institution where a dominant number of the sample consisted of Malay-ethnicity participants, which limits external generalisation to other Southeast Asian collegiate cohorts and to multi-ethnic athletes.

This study carries two main implications for sport scientists and pathway practitioners working with collegiate-tier Southeast Asian athletes. The present data argue against using birth quartile as a proxy for biomechanical loading risk in athletes who have already entered pathway-level competition; coaches and medical staff should not adjust screening priority on the basis of relative age in this population. Additionally, the demonstrable male RAE (Q1:Q4 = 2.06) suggests the selection system continues to filter for relatively older athletes, with the implication that talent-identification programmes operating within this cohort should consider bio-banded or maturity-aware adjustments at earlier stages of the pathway, before the survivor cohort reaches the present sample's level (47, 48). The quantitative corrective adjustment procedures which have already been validated for swimming and athletics provide a complementary mechanism by which pathway organisations can statistically rebalance early-stage selection without disrupting existing competitive structures (49).

Future work should test the survivorship interpretation prospectively by tracking clinical injury incidence in a cohort screened at entry to the pathway, with birth quartile as a covariate (12). This design would distinguish whether the null cross-sectional finding reflects genuine biomechanical-loading equivalence, selective attrition of biomechanically vulnerable Q4 athletes prior to assessment, or insufficient sensitivity of the modelled loading estimates to capture clinically relevant injury-risk variation. More broadly, a recent scoping review has documented that the athlete development literature, which encompasses the relative age effect, and the athlete health literature, which covers injury and biomechanical research, have developed largely independently, with explicit calls for cross-disciplinary integration (50). Continued empirical work bridging these two literatures, which the present study attempts, is warranted to determine whether the survivorship pattern observed here generalises across populations, prospective designs, and independently validated measurement frameworks.

## 5 Conclusions

In the cohort of Malaysian university athletes, a significant relative age effect was identified at the cohort level and in male athletes, but no AI-derived biomechanical loading-risk index differed across birth quartiles in either continuous or binary analyses. The selection-related birthdate asymmetry was therefore present without detectable biomechanical-loading consequences, a pattern consistent with a survivorship interpretation. Birth quartile should not be used as a proxy for biomechanical loading risk in athletes who have already entered collegiate-level competition. Prospective injury surveillance is required to test whether the observed equivalence reflects genuine biomechanical-loading robustness, selective attrition of vulnerable late-born athletes upstream of the screening point, or limitations of cross-sectional AI-derived loading estimation in capturing clinical injury risk.

## Data Availability

The original contributions presented in the study are included in the article/Supplementary Material, further inquiries can be directed to the corresponding author/s.
